# Rise and Growth of Vaccination Law.—III

**Published:** 1903-11-07

**Authors:** J. E. R. Stephens

**Affiliations:** Barrister-at-law, author of "Digest of Public Health Cases," etc., etc.


### RISE AND GROWTH OF VACCINATION LAW.—III.

By J. E. R. Stephens, Barrister-at-law, author of "Digest of Public Health Cases," etc., etc.

(*Continued from page* 39.)

On May 23rd, 1871, the Select Committee on Vaccination reported (Sess. Pap. No. 246) to the House as follows:—

That the cow-pox affords, if not an absolute, yet a very great protection against an attack of small-pox, and an almost absolute protection against death from that disease. That if the operation be performed with due regard to the health of the person vaccinated, and with proper precautions in obtaining and using the vaccine lymph, there need be no apprehension that vaccination will injure health or communicate any disease. That small-pox unchecked by vaccination is one of the most terrible and destructive of diseases, as regards the danger of infection, the proportion of deaths among those attacked, and the permanent injury to the survivors; and therefore that it is the duty of the State to endeavour to secure the careful vaccination of the whole population. The Committee have no doubt that the almost universal opinion of medical science and authority is in accordance with Dr. Gull when he states that "vaccination is as protective against small-pox as small-pox itself"; with Dr. West when he states as the result of his experience as physician to the Children's Hospital in Great Ormond Street, and as having had charge of between 50,000 and 60,000 children since 1835, that "he does not think that vaccination does produce disease"; and with Sir William Jenner when he says, "I should think myself wicked and really guilty of a crime if I did not recommend every parent to have his child vaccinated early in life." Against this evidence in favour of vaccination, the prevalence of the present (*i.e.* 1871) small-pox epidemic, especially in the metropolis, has been alleged. The Committee, however, believe that on the other hand if vaccination had not been general, this epidemic might have become a pestilence as destructive as small-pox has often been, where the population has been unprotected, and that on the other hand if this preventive had been universal the epidemic could not have approached its present extent. Vaccination is generally believed to require repetition about the age of puberty, but as it is almost impossible to enforce revaccination, it is most important that all children should be vaccinated, both for their own sakes and that of the community, to prevent their catching and spreading disease.

There are three classes of children who, being by the conduct of their parents left unvaccinated, are themselves in great danger, and may become centres of infection to others. 1. There are the children who are utterly neglected by their parents. 2. There is the much larger number of children of parents who, while not denying their duty or desiring to disregard it, postpone its fulfilment, and who from carelessness and forgetfulness delay to protect their children until driven to the vaccine station by the panic fear of an epidemic. 3. There are children of those parents, very few in proportion to the whole population, who assert that vaccination will do harm. With regard to the first and second of these classes there can hardly be any objection to the principle of a compulsory law, though there may be practical difficulties in its application; but in dealing with the third class it becomes necessary to weigh the claims of the parent to control, as he thinks fit, the medical treatment of an infant child, as against the duty of the State to protect the health of the community, and to save the child itself from a dreadful disease.

While weighing these conflicting claims the committee had to consider the effect of the change in the law introduced by the Act of 1867, which, contrary to the provisions of the previous Acts, makes the parent liable to repeated convictions and penalties for not allowing his child to be vaccinated. There appear to have been several cases of infliction of more than one fine or imprisonment in regard to the same child, and the committee, though by no means admitting the right of the parent to expose his child or his neighbours to the risk of small-pox, must express great doubt whether the object of the law is gained by thus continuing a long contest with the convictions of the parent. The public opinion of the neighbourhood may sympathise with a person thus prosecuted, and may in consequence be excited against the law; and after all, though the parent be fined or imprisoned, the child may remain unvaccinated. In such a case the law can only triumph by the forcible vaccination of the child. In enactments of this nature, when the State, in attempting to fulfil the duty, finds it necessary to disregard the wish of the parent, it is most important to secure the support of public opinion, and, as the committee cannot recommend that a policeman should be empowered to take a baby from its mother to the vaccine station—a measure which could only be justified by an extreme necessity—they would recommend that, whenever in any case two penalties, or one full penalty, have been imposed upon a parent, the magistrate should not impose any further penalty in respect of the same child. It has been suggested that the parent's declaration of belief that vaccination is injurious might be pleaded against any penalty; but the committee believe that if the law were thus changed it would become a dead letter. Prosecutions would soon cease, and the children of the many apathetic and neglectful parents would be left unvaccinated, as well as the children of the few opponents of vaccination. The Committee are strongly of opinion that the registration of vaccination should be simplified; that the vaccination officer should keep the vaccination register, and therefore that the certificates under the Act should be sent to him, and also that the registrar of the district should forward to him a monthly return of births, and of the infants that have died.

On June 12th, 1871, Mr. Forster, on behalf of the Government, brought in a Bill "to amend the Vaccination Act of 1867," in which the recommendations of the Committee were embodied, and on August 21st, 1871, the Act, 34 and 35 Vict. c. 98, received the Royal asent. The Bill introduced by Mr. Forster contained a clause which was founded on the recommendation of the Committee as to the limitation of the penalties, which was as follows:—

"After the commencement of this Act, no parent of a child shall be liable to be convicted for neglecting to take, or to cause to be taken, such child to be vaccinated, or for disobedience to any order directing such child to be vaccinated, if either—(*a*) he has been previously adjudged to pay the full penalty of 20s. for any of such offences with respect to such child, or (*b*) he has been previously twice adjudged to pay any penalty for any such offences in respect of such child."

This clause was rejected by the House of Lords by a majority of one, and the Bill received the Royal assent without it. In the next session a Bill containing a similar clause was brought in by Mr. Pease, but was withdrawn.

Much difficulty has been experienced by those who have had to administer the vaccination laws in determining how far it is desirable to repeat proceedings against defaulters. On the one hand it was felt that the intention of the Acts would be defeated if, by the payment of a fine of 20s., a person could purchase exemption from vaccination, and on the other, it was desired not to deal harshly with persons who really held conscientious objection to the vaccination of their children. With a view to assist boards of guardians in the matter, the Local Government Board published a letter which, in 1875, they addressed to the guardians of the Evesham Union. That letter advised guardians to carefully consider, with regard to each individual case, the effect which a continuance of proceedings was likely to have in procuring the vaccination of the individual child, and in insuring the observance of the law in the union generally.

*(To be continued.)*

